# Fulminant disseminated carcinomatosis of the bone marrow from pancreatic mucinous carcinoma with predominant signet ring cell morphology

**DOI:** 10.1007/s12328-026-02332-1

**Published:** 2026-04-17

**Authors:** Katsuya Ami, Keiko Kamei, Masaya Nakano, Chihoko Nobori, Yuta Yoshida, Kentaro Tai, Takaaki Murase, Atsushi Takebe, Takaaki Chikugo, Ippei Matsumoto

**Affiliations:** 1 Division of Hepato-Biliary-Pancreatic SurgeryDepartment of SurgeryFaculty of Medicine, Kindai University, 1-14-1 Mihara-Dai, Minami-Ku, Sakai, Osaka 590-0197 Japan; 2 Department of Diagnostic Pathology, Faculty of Medicine, Kindai University, 1-14-1 Mihara-Dai, Minami-Ku, Sakai, Osaka 590-0197 Japan

**Keywords:** Disseminated carcinomatosis of the bone marrow, Pancreatic cancer, Pancreatic mucinous carcinoma, Signet ring cell carcinoma, Disseminated intravascular coagulation

## Abstract

Disseminated carcinomatosis of the bone marrow (DCBM) is a rare and highly aggressive form of bone metastasis arising from solid tumors, most commonly gastric cancers. DCBM originating from pancreatic cancer is exceedingly rare, with only a few cases reported in the literature. A 62-year-old man presented with back pain, loss of appetite, and weight loss. Laboratory tests revealed anemia and thrombocytopenia. Contrast-enhanced computed tomography revealed a hypovascular mass measuring approximately 10 mm in size in the pancreatic tail. Endoscopic ultrasound-guided fine-needle aspiration confirmed the diagnosis of pancreatic mucinous carcinoma. Laboratory evaluation suggested disseminated intravascular coagulation (DIC). Although no nodular bone metastases were detected on imaging studies, bone marrow biopsy revealed diffuse infiltration by adenocarcinoma predominantly composed of tumor cells with signet ring cell carcinoma morphology, leading to a diagnosis of DCBM from pancreatic mucinous carcinoma. The patient developed a subarachnoid hemorrhage due to severe bleeding tendency, and chemotherapy could not be initiated. The patient died on hospital day 11. To our knowledge, this is the first reported case of DCBM arising from pancreatic mucinous carcinoma with bone marrow metastasis predominantly composed of signet ring cell carcinoma morphology. In patients with pancreatic cancer who present with DIC, particularly mucin-producing tumors such as mucinous carcinoma, the possibility of DCBM should be actively suspected, and early bone marrow aspiration and biopsy should be considered.

## Introduction

Disseminated carcinomatosis of the bone marrow (DCBM) is a rare and highly aggressive subtype of bone metastasis originating from solid tumors and is characterized by diffuse infiltration of cancer cells throughout the bone marrow cavity without the formation of discrete nodular lesions. DCBM is frequently associated with hematological abnormalities such as disseminated intravascular coagulation (DIC) and hemolytic anemia, and its prognosis is considered extremely poor [1, 2]. In most cases, the primary tumor is gastric cancer (90%), followed by colorectal cancer, lung cancer, and breast cancer [3]; however, metastasis from pancreatic cancer is exceedingly rare. Herein, we report the case of a patient with DCBM arising from pancreatic mucinous carcinoma with bone marrow metastasis predominantly composed of tumor cells showing signet ring cell carcinoma morphology, with DIC and hemolytic anemia that exhibited a rapidly progressive clinical course.

## Case report

**Fig. 1:**
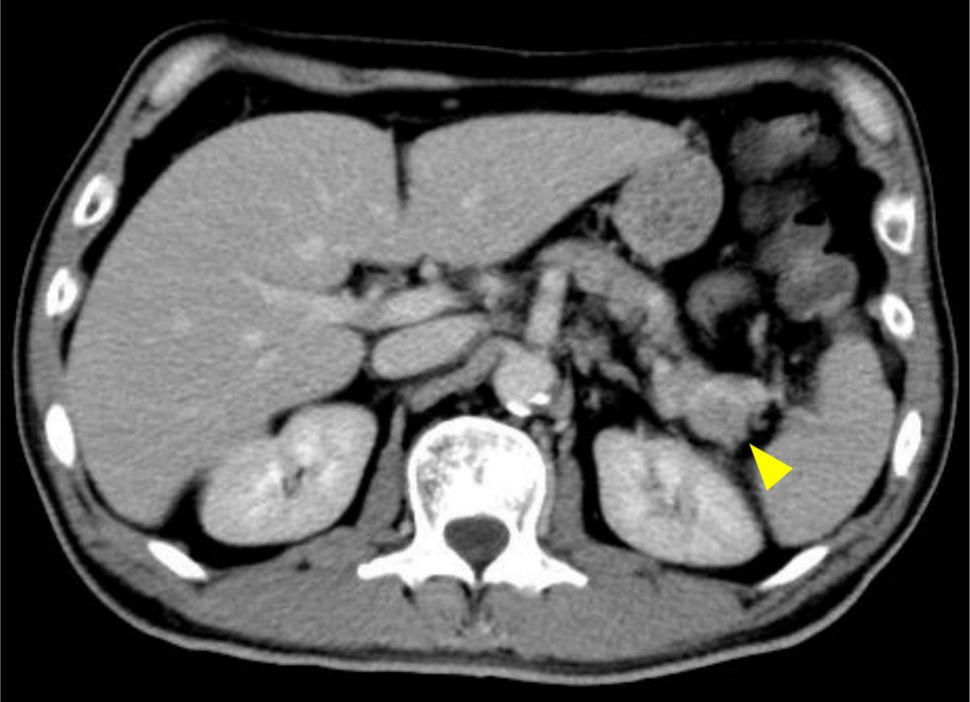
Contrast-enhanced computed tomography of the chest and abdomen performed at the referring hospital revealed a 10 mm hypovascular mass in the pancreatic tail (yellow arrowhead). No evidence of distant metastases, including nodular bone metastases, or other findings suggestive of malignancy was observed

**Fig. 2:**
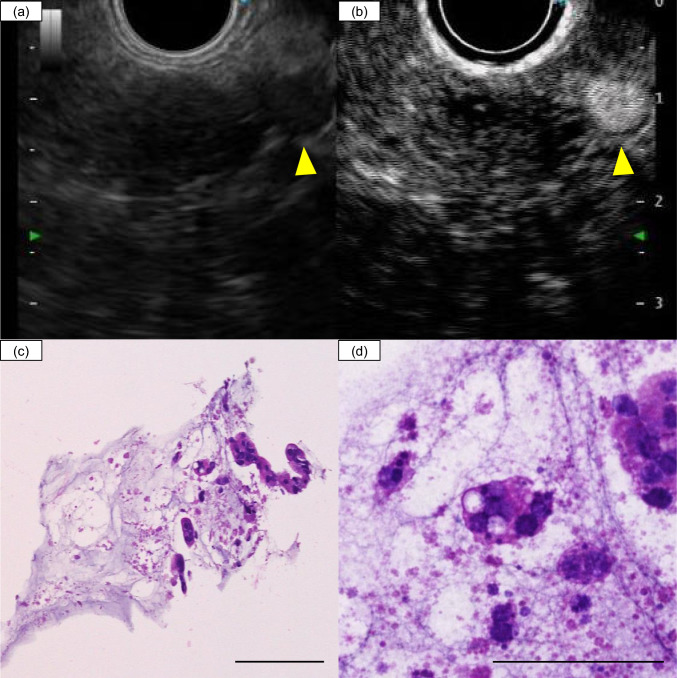
Endoscopic ultrasound (EUS) demonstrated a 10-mm hypoechoic mass in the pancreatic tail (**a**). Contrast-enhanced harmonic EUS using perflubutane microbubbles (Sonazoid®, GE Healthcare) showed distinct hyperenhancement of the tumor (**b**). Histopathological examination of EUS-guided fine-needle aspiration specimens revealed adenocarcinoma with abundant extracellular mucin, consistent with mucinous carcinoma (hematoxylin and eosin staining) (**c**). Higher-magnification views showed tumor cells with intracytoplasmic mucin accumulation within the mucinous background (**d**). Scale bars indicate 100 µm (**c**), 50 µm (**d**)

**Fig. 3:**
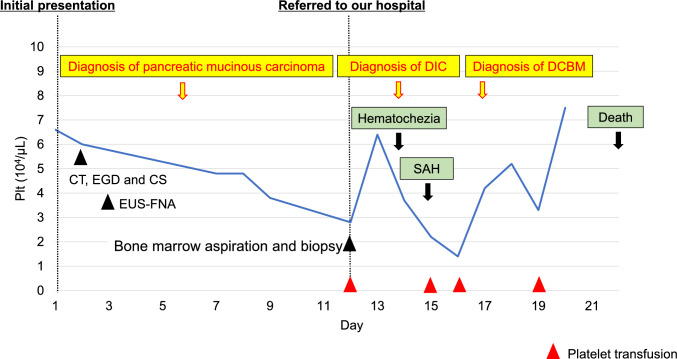
Temporal changes in platelet count with key clinical events at the referring hospital and our institution. CS, colonoscopy; CT, computed tomography; DCBM, disseminated carcinomatosis of the bone marrow; DIC, disseminated intravascular coagulation; EGD, esophagogastroduodenoscopy; EUS-FNA, endoscopic ultrasound-guided fine-needle aspiration; Plt, platelet count; SAH, subarachnoid hemorrhage

**Fig. 4:**
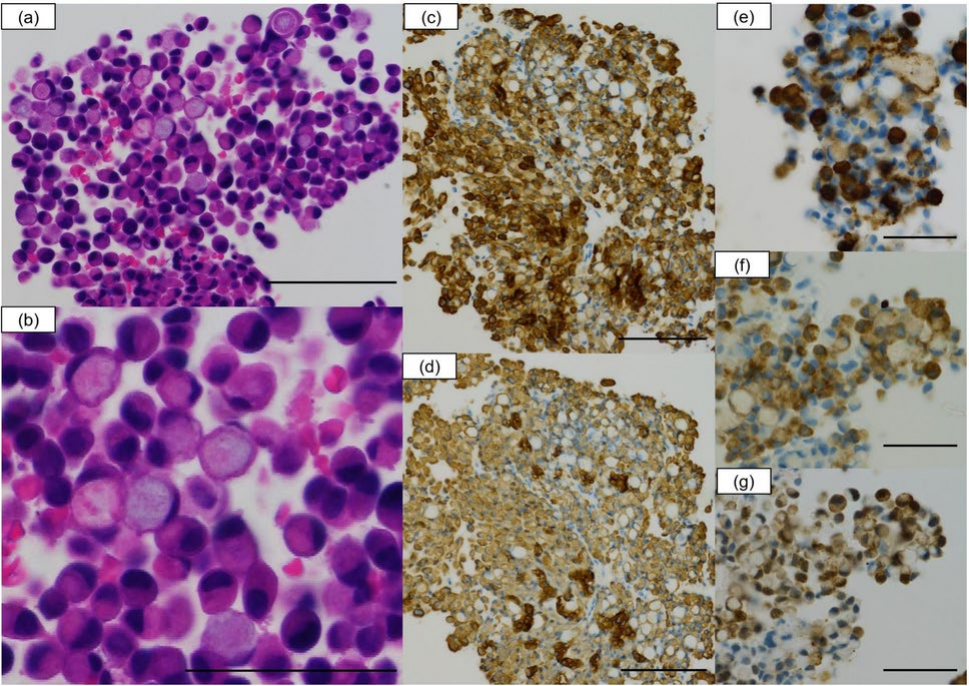
Histopathological examination of the bone marrow biopsy specimen revealed diffuse infiltration of adenocarcinoma predominantly composed of tumor cells showing signet ring cell carcinoma morphology (hematoxylin and eosin staining) (**a**, **b**). Immunohistochemical staining demonstrated positivity for CK7 (**c**), CK20 (**d**), CA19-9 (**e**), IMP3 (**f**), and maspin (**g**). Scale bars indicate 100 µm (**a**, **c**, **d**), 50 µm (**b**, **e**, **f**, **g**)

A 62-year-old man with no significant medical history presented to his primary physician with a two-week history of back pain, loss of appetite, and weight loss. Laboratory investigations performed at the referring hospital revealed jaundice, anemia, and thrombocytopenia. Abdominal contrast-enhanced computed tomography (CT) revealed a hypovascular mass in the pancreatic tail (Fig. 1). Endoscopic ultrasonography (EUS) with contrast enhancement using perflubutane microbubbles (Sonazoid®, GE Healthcare) demonstrated a hyperenhanced lesion measuring up to 10 mm in size in the pancreatic tail (Fig. 2a, b). EUS-guided fine-needle aspiration (EUS-FNA) revealed a mucinous carcinoma, leading to a diagnosis of pancreatic mucinous carcinoma. Although mucus accumulation was observed within the cytoplasm of tumor cells, typical signet ring cell morphology was not identified (Fig. 2c, d). Neither upper nor lower gastrointestinal endoscopy revealed gross lesions suggestive of malignancy. The patient was referred to our hospital 11 days after his initial visit to the previous physician because the anemia had progressed to a level requiring blood transfusion. The clinical course, including the timing of key investigations at the referring hospital and our institution, is summarized in Fig. 3. Consciousness was mildly drowsy, with a Glasgow Coma Scale score of E4V4M6. His body temperature was 38.0 °C, blood pressure was 134/75 mmHg, and pulse was 94 beats per minute. His height, weight, and body mass index were 172.9 cm, 63.4 kg, and 21.2 kg/m2, respectively. An abdominal examination revealed mild back pain. There was no relevant family history. The laboratory data at the time of presentation to our hospital are shown in Table 1. The findings revealed anemia and thrombocytopenia. Elevated levels of the inflammatory markers, lactate dehydrogenase, alkaline phosphatase, and bilirubin, with a predominance of indirect bilirubin, were observed. The routine coagulation test results were not markedly abnormal; however, an elevated D-dimer level was observed. Elevated levels of Thrombin-Antithrombin Complex and Plasmin-α2 Plasmin Inhibitor Complex were observed. These findings suggest the presence of DIC, which is characterized by the activation of both the coagulation and fibrinolysis pathways. Additionally, tumor markers, including carcinoembryonic antigen, carbohydrate antigen 19–9 (CA19-9), Duke pancreatic monoclonal antigen type 2 (DUPAN-2), and S-pancreas-1 antigen (SPan-1), were elevated. Chest and abdominal CT revealed a 10-mm tumor in the tail of the pancreas and a branch-duct-type intraductal papillary mucinous neoplasm in the head of the pancreas. No distant metastases, including nodular bone metastases, were observed. Bone marrow aspiration was performed; however, because of a dry tap with no bone marrow fluid, a bone marrow biopsy was performed. Histopathological examination revealed diffuse infiltration of adenocarcinoma predominantly composed of tumor cells, showing signet ring cell carcinoma morphology on hematoxylin and eosin staining (Fig. 4a, b). Immunohistochemical staining revealed that the atypical cells were positive for AE1/AE3, CK7, CK19, CK20, CA19-9, IMP3, and maspin (Fig. 4c–g). Based on these findings, a diagnosis of DCBM from pancreatic mucinous carcinoma with bone marrow metastasis, predominantly composed of signet ring cell carcinoma, was established.

**Table 1:** Laboratory findings at the time of transfer to our hospital

WBC	11,260	/uL	T-bil	3.9	mg/dL	PT (sec)	13.7	sec
RBC	141	10^4^/uL	D-bil	1.4	mg/dL	PT (%)	67.2	%
Hb	4.7	g/dL	I-bil	2.5	mg/dL	PT (INR)	1.21	
Plt	2.8	10^4^/uL	AMY	31	U/L	APTT (sec)	31.0	sec
TP	6.6	g/dL	LDH	1,509	U/L	Fib	160	mg/dL
Alb	3.7	g/dL	CPK	202	U/L	AT-III	99	%
Na	141	mmol/L	BUN	35	mg/dL	TAT	47.9	ng/mL
K	3.9	mmol/L	Cre	0.92	mg/dL	FDP	79.9	µg/mL
Cl	102	mmol/L	Glu	122	mg/dL	D-dimer	34.8	µg/mL
Ca	9.3	mg/dL	CRP	6.79	mg/dL	PIC	12.5	µg/mL
AST	74	U/L				CEA	15.0	ng/mL
ALT	34	U/L				CA19-9	191	U/mL
ALP	1,236	U/L				DUPAN-2	3,375	U/mL
γ-GTP	23	U/L				SPan-1	1,551	U/mL

*WBC* white blood cell, *RBC* red blood cell, *Hb* hemoglobin, *Plt* platelet, *TP* total protein, *Alb* albumin,

*Na* sodium, *K* potassium, *Cl* chloride, *AST* aspartate aminotransferase, *ALT* alanine aminotransferase, *ALP* alkaline phosphatase, *γ-GTP* γ-glutamyl transpeptidase, *T-Bil* total bilirubin, *D-Bil* direct bilirubin, *I-Bil* indirect bilirubin, *AMY* amylase, *LDH* lactate dehydrogenase, *CPK* creatine phosphokinase, *BUN* blood urea nitrogen, *Cre* creatinine, *Glu* glucose, *CRP* C-reactive protein, *PT* prothrombin time, *sec* second, *INR* international normalized ratio, *APTT* activated partial thromboplastin time, *Fib* fibrinogen, *AT-III* antithrombin, *TAT* thrombin-antithrombin complex, *FDP* fibrin degradation products, *PIC* plasmin-α2 plasmin inhibitor complex, *CEA* carcinoembryonic antigen, *CA19-9* carbohydrate antigen 19–9, *DUPAN-2* Duke pancreatic monoclonal antigen type 2, *SPan-1* S-pancreas-1 antigen

The patient exhibited worsening hemolytic anemia and thrombocytopenia associated with DIC, necessitating red blood cell and platelet transfusions. DIC was clinically recognized on hospital day 3; however, hematochezia, reflecting a progressive systemic bleeding tendency, had already developed by that time, precluding the initiation of treatment. On hospital day 4, the patient developed impaired consciousness, and head CT revealed a subarachnoid hemorrhage. The bleeding tendency persisted, and the overall condition did not improve. The patient died on hospital day 11, which was 22 days after the initial presentation.

## Discussion

DIC associated with diffuse bone metastases of solid tumors was first reported by Jarcho et al. in 1936 [4]. In Japan, Hayashi et al. defined DCBM as a condition characterized by diffuse organ infiltration, primarily in the bone marrow, due to extensive hematogenous and lymphatic spread, accompanied by hemorrhagic symptoms caused by DIC and hemolytic anemia [1]. Most cases are metastases from gastric cancer, followed by colon, lung, and breast cancer [3]. DCBM originating from pancreatic cancer is extremely rare, with only two cases reported to date, both of which showed rapid clinical deterioration and extremely poor prognoses (Table 2) [5, 6]. Furthermore, no cases of pancreatic mucinous carcinoma with bone marrow metastasis predominantly composed of signet ring cell carcinoma have been previously reported, making this the first documented case in the literature.

**Table 2:** Reported cases of disseminated carcinomatosis of the bone marrow arising from pancreatic cancer

Case No	1	2	3
Author (year)	Nakamura (2012)	Namikawa (2016)	Our case (2026)
Age / Sex	72 / Male	57 / Male	62 / Male
Symptoms	Constipation, urinary frequency, back pain	Severe lumbago	Back pain, appetite loss, weight loss
DIC	- (developed after chemotherapy)	+	+
Tumor location / Size	Pancreatic tail / 55 mm	Pancreatic tail / 28 mm	Pancreatic tail / 10 mm
Histology	Adenocarcinoma	Poorly differentiated adenocarcinoma	Mucinous carcinoma
Method of pancreatic cancer diagnosis	PET-CT, EUS-FNA	PET-CT, CE-CT	CE-CT, EUS-FNA
Method of bone marrow diagnosis	Bone marrow biopsy	Bone marrow biopsy	Bone marrow biopsy
Treatment	Gemcitabine → Gemcitabine + S-1 and DIC management	Supportive care and DIC management	Supportive care
Outcome	Died 82 days after treatment initiation	Died approximately 60 days after admission	Died on hospital day 11 (22 days after the initial presentation)

*DIC* disseminated intravascular coagulation, *EUS-FNA* endoscopic ultrasound–guided fine-needle aspiration, *PET-CT* positron emission tomography–computed tomography, *CE-CT* contrast-enhanced computed tomography

Mucin-producing adenocarcinomas increase the risk of cancer-associated DIC through tumor-derived mucins and procoagulant factors that activate the coagulation cascade [7, 8]. Furthermore, mucinous carcinomas may contain tumor cells with signet ring cell morphology within pools of extracellular mucin, which may not be fully represented in limited biopsy specimens. In the present case, EUS-FNA of the primary pancreatic lesion revealed a mucinous carcinoma, whereas the metastatic lesions within the bone marrow were predominantly composed of tumor cells with signet ring cell carcinoma morphology, resulting in DCBM. In the present case, the EUS-FNA specimens of the primary tumor were re-evaluated after the diagnosis of bone marrow metastasis. Although intracytoplasmic mucin accumulation was observed in tumor cells, typical signet ring cell carcinoma morphology was not identified. Therefore, the morphological discrepancy between the primary tumor and metastatic lesions may be explained, at least in part, by sampling limitations inherent to EUS-FNA [9]. This discrepancy between the primary tumor and metastatic sites suggests intratumoral heterogeneity, preferential survival, and expansion of tumor cell populations with signet ring cell carcinoma features during systemic dissemination [10, 11]. Across organ systems, the signet ring cell carcinoma morphology is characterized by discohesive growth with reduced cell–cell adhesion, which predisposes tumor cells to single-cell infiltration and microvascular invasion [12, 13]. Moreover, the predominantly hematogenous pattern of spread associated with this morphology may facilitate tumor cell seeding within the bone marrow sinusoids, leading to diffuse marrow infiltration rather than nodular bone metastases [14]. These biological characteristics may explain why tumor cells showing signet ring cell carcinoma morphology, even when only focally present in the primary tumor, preferentially expand within the unique microenvironment of the bone marrow in the present case. Therefore, in patients with pancreatic cancer, particularly mucin-producing adenocarcinomas such as mucinous carcinoma who present with DIC, the possibility of DCBM should be considered, and early bone marrow aspiration and biopsy should be pursued for a definitive diagnosis.

In the present case, contrast-enhanced CT revealed a hypovascular mass measuring approximately 10 mm in the pancreatic tail. EUS-FNA confirmed the diagnosis of pancreatic mucinous carcinoma. Furthermore, laboratory findings demonstrated markedly elevated levels of SPan-1 and DUPAN-2, as well as the presence of DIC. Bone marrow biopsy demonstrated diffuse infiltration predominantly composed of tumor cells showing signet ring cell carcinoma morphology, leading to a diagnosis of DCBM from pancreatic mucinous carcinoma. Although random gastric biopsies were not performed, both upper and lower gastrointestinal endoscopy revealed no abnormal findings, and a gastrointestinal primary tumor, including occult gastric cancer, was considered unlikely. Although the immunohistochemical markers used in this case are not entirely specific for pancreatic origin, the overall staining pattern, in combination with the clinical and imaging findings, was considered most consistent with a pancreatic primary. Notably, despite the relatively small size of the primary tumor compared with previously reported cases, DCBM had already developed at the time of diagnosis, and its clinical course was characterized by extremely rapid disease progression. This observation suggests that, in aggressive pancreatic malignancies, tumor size does not necessarily correlate with biological aggressiveness or tumor burden. Mucin-producing adenocarcinomas, including mucinous carcinoma, are known to exhibit aggressive biological behavior, partly due to tumor-derived mucins that facilitate hematogenous dissemination and thrombus formation [7, 8]. Signet ring cell carcinoma morphology has also been associated with a higher propensity for diffuse infiltration and early systemic spread in several organ systems [12, 13]. However, evidence specifically linking these features to rapid bone marrow dissemination in pancreatic cancer remains limited, and further accumulation of cases is required. Although data regarding the diagnostic performance for pancreatic cancers measuring ≤ 10 mm are limited, previous studies have indicated that EUS may achieve a higher detection yield than other imaging modalities [15, 16], whereas the diagnostic accuracy of EUS-FNA tends to decline in sub-centimeter lesions [9]. Therefore, even small pancreatic lesions may represent biologically aggressive disease, and careful multimodal evaluation using contrast-enhanced CT, magnetic resonance imaging, and EUS is important [17].

DCBM is an extremely aggressive condition associated with poor prognosis, with a median overall survival of approximately 1–3 months in patients with gastric cancer [14, 18]. Despite this, previous studies have suggested that survival may be prolonged in patients who receive systemic chemotherapy compared to those managed with best supportive care alone [18]. At present, no standard chemotherapy regimen has been established for pancreatic cancer with DCBM; however, in treatment strategies for metastatic pancreatic cancer, intensive combination chemotherapy, such as gemcitabine plus nab-paclitaxel or FOLFIRINOX, has been reported to be associated with prolonged survival [19, 20]. In contrast, patients with DCBM frequently present with DIC and profound bone marrow suppression, which often precludes the initiation of systemic chemotherapy. In the present case, severe anemia and a bleeding tendency associated with DIC led to the development of a subarachnoid hemorrhage, and the rapid deterioration of the patient’s general condition rendered chemotherapy infeasible. A short but critical interval elapsed between the patient’s initial presentation at the referral hospital and the definitive diagnosis of DCBM from pancreatic mucinous carcinoma at our institution. The opportunity for therapeutic intervention was markedly limited at the time of diagnosis. These findings indicate that in patients with DCBM, early diagnosis and prompt intervention for DIC are crucial for stabilizing the patient’s condition and preserving the possibility of initiating systemic chemotherapy. Although DIC associated with DCBM is not clearly distinguishable from conventional cancer-associated DIC in terms of formal diagnostic criteria, it is often characterized by rapidly progressive anemia and thrombocytopenia, markedly elevated lactate dehydrogenase and alkaline phosphatase levels, and severe hematological abnormalities disproportionate to imaging findings. In such cases, early diagnosis with a high index of suspicion for DCBM is essential [2].

In conclusion, this report describes the first documented case of DCBM arising from pancreatic mucinous carcinoma in which the bone marrow metastatic lesions were predominantly composed of tumor cells with a signet ring cell carcinoma morphology. Despite the small size of the primary tumor, the disease exhibits highly aggressive biological behavior with early systemic dissemination, including DCBM. In patients with suspected pancreatic cancer accompanied by DIC, clinicians should consider the possibility of DCBM, pursue early diagnostic evaluation, and provide prompt intervention to stabilize the patient’s condition and preserve treatment opportunities.
